# Association of the *ATG9B * gene polymorphisms with coronary artery disease susceptibility: A case-control study

**DOI:** 10.15171/jcvtr.2019.19

**Published:** 2019-06-25

**Authors:** Mahsa Mehrabi Pour, Mahboobeh Nasiri, Hajar Kamfiroozie, Mohammad Javad Zibaeenezhad

**Affiliations:** ^1^Department of Biology, Islamic Azad University, Arsanjan Branch, Arsanjan, Iran; ^2^Cardiovascular Research Center, Shiraz University of Medical Sciences, Shiraz, Iran

**Keywords:** Coronary Artery Disease, *ATG9B*, Polymorphism

## Abstract

***Introduction:*** Endothelial nitric oxide synthase (eNOS), the main regulator of cardiac cell functioning, is regulated post-transcriptionally by autophagy-related 9B (*ATG9B*) gene. The proper function of the heart is partly determined by the intact interaction of these molecules. The present study aimed to investigate the effects of ATG9B rs2373929 and rs7830 gene polymorphisms on the predisposition to coronary artery disease (CAD).

***Methods:*** In this hospital-based case-control study, 150 patients with CAD compared with 150 healthy subjects for the genotype distributions of rs2373929 and rs7830 polymorphisms using T-ARMS PCR and ARMS PCR, respectively.

***Results:*** Considering rs2373929 polymorphism, increased risk of CAD observed in the presence of TT genotype (OR: 3.65; 95% CI: 1.77-7.53; *P *< 0.001) and also in the recessive model for T allele (OR: 3.41; 95% CI: 1.76- 6.60; *P *< 0.001). The frequency of the T allele was higher in cases compared to controls (OR: 1.71; 95% CI: 1.24-2.28; P = 0.001). The genotype and allele frequencies of the rs7830 polymorphism did not differ between the two study groups.

***Conclusion:*** The *ATG9B * gene rs2373929 polymorphism might involve in the pathogenesis of the CAD and can be considered as a screening molecular marker in the subjects prone to CAD.

## Introduction

Coronary artery diseases (CADs) categorize as a group of cardiovascular disorders characterized by low blood and oxygen supply of the heart muscle mainly due to plaque formation (atherosclerosis) on the inner wall of the coronary arteries.^1^ Older age, male gender, family history of heart disease, hypercholesterolemia, and high blood pressure, previous history of diabetes mellitus, obesity, smoking habit, and genetic background are forming a complex combination of environmental and genetic factors predisposing people to CAD.^2,3^

A new generation of molecular technologies such as high-throughput RNA sequencing enhances our insight about the heterogeneity of the transcriptome; indeed only a very low fraction (2%) of the whole RNA transcripts of a cell has the protein-coding potential.^4,5^ Long non-coding RNAs (lncRNAs) with the length of 200 nucleotides to 100 kilobases and diverse regulatory capacity are the newly described part of this heterogeneity.^6^ LncRNAs are involved in numerous important biological phenomena such as the regulation of imprinting loci, gene expression, and chromatin organization.^7,8^ An aberration in the lncRNA gene expression profile and their nucleotide variants are implicated to be involved in many human tumorigenesis pathways and other diseases such as CAD.^9,10^ This area of molecular medicine is in its infancy and further investigation needs to provide detailed information regarding the importance of lncRNAs in disease pathogenesis.

Autophagy-related 9B (*ATG9B or SONE*) is a protein-coding gene playing role in lysosomal degradation pathway, but it also functions as an overlapping cis-antisense transcript complementary to the 3′UTR region of endothelial nitric oxide synthase (eNOS) mRNA.^11^ Endothelial cells characterized by high levels of eNOS mRNA and protein. Therefore, measuring high levels of *ATG9B* in vascular smooth muscle cells (VSMCs) but the lower levels in endothelial cells confirms its role as an antisense transcript for eNOS.^11^ In hypoxic endothelial cells, the *ATG9B* mRNA half-life increases and in turn the level of eNOS significantly falls.^12^ This reciprocal expression pattern along with the results of RNA-interference-mediated knockdown of *ATG9B* in VSMC highlighted the role of *ATG9B* as a post-transcriptionally negative regulator of eNOS.^12^ The health of the vascular endothelium is guaranteed by the precise production of nitric oxide (NO) maintaining the vascular tone and cardiac contractility.^13^ Any changes in the amount of NO result in vascular endothelial injury which in turn predispose them to coronary artery atherosclerosis.^14^ NO synthesis is mainly regulated by the eNOS enzyme.^15^ Pathophysiological stimuli such as shearing stress, hypoxia, LDL oxidation, and inflammatory cytokines influenced the stability and function of eNOS in endothelial cells.^16^ Any alteration in these complex molecular interactions may play a role in the pathogenesis of CAD. Plus the fact that single nucleotide polymorphisms (SNPs) are the most prevalent tandem repeated sequences distributed over the genome, they amplified easily using PCR-based methods. ^17^ On account of the above reasons, SNPs seem like the best DNA markers for screening the susceptibility loci for multifactorial diseases. ^17^

Although some researchers have recently reported the association of lncRNA variants with CAD,^18^ whether the variants of *ATG9B* gene affect the susceptibility to CAD is not clear. The purpose of this study was to evaluate the association between *ATG9B* rs2373929 and rs7830 intronic polymorphisms and susceptibility to CAD in a population from southwest Iran.

## Materials and Methods

### Characteristics of the study subjects

A total of 150 high-risk patients with CAD diagnosed at Al-Zahra Heart Center, Shiraz, Iran was recruited as a case group in this hospital-based case-control study. Patients were diagnosed by an expert cardiologist among the ones hospitalized in angiography ward according to the presence of angiographically documented atherosclerotic lesions in coronary vessels. The inclusion criteria were; age beyond 45 years for men, and 55 years for women, the presence of at least one of the risk factors for CAD including; cigarette smoking, hypercholesterolemia, diabetes mellitus (DM), hypertension, and family history of CAD in first-degree relatives. Current smokers were considered as a positive smoking status, and who never smoke as a negative group. Hypercholesterolemia was considered as either under treatment with lipid-lowering drugs or having total cholesterol greater than 200 mg/dL. Blood pressure greater than 140/90 mm Hg or even being on therapy considered as hypertension. Diabetes mellitus was diagnosed with fasting blood glucose level >126 mg/dL or dependency on insulin or other hypoglycemic drugs. If there was more than one patient in the family, only one recruited to the study and the others were excluded. The control group subjects (150 samples) were selected from healthy blood donors based on not having the previous history of CAD or any other heart disorders and matched on gender and age.

### Data collection

A self-prepared questionnaire considering demographic and anthropometric information, cigarette smoking, history of diabetes, hypertension, hypercholesterolemia, and family history of CAD in first-degree relatives was filled by the knowledgeable nurses prior to blood sampling. The subject with at least one first-degree relative with CAD is considered to have a positive family history. All parts of the procedure were supported by the Institutional Review Board at our Department.

### Molecular genotyping

**Figure 1:**
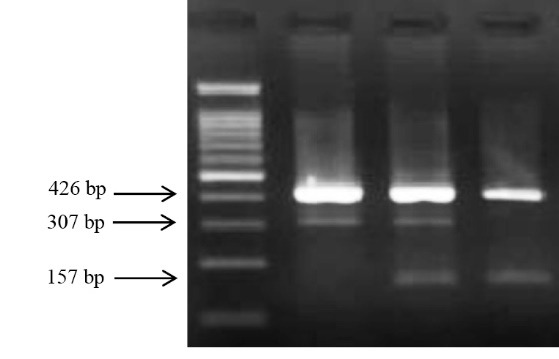
The electrophoresis pattern of the different genotypes of the rs2373929 following T-ARMS PCR. From left to right: 100 bp ladder, TT homozygous, CT heterozygous, and CC homozygous genotype.

**Figure 2:**
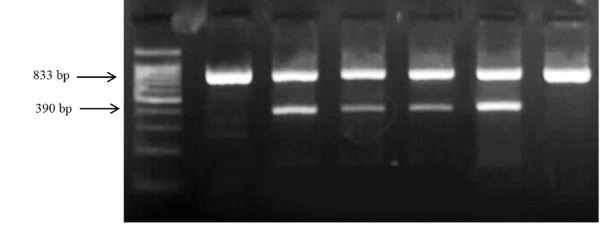
The electrophoresis pattern of the different genotypes of the rs7830 following ARMS PCR. From left to right: 100 bp ladder, CC homozygous, AC heterozygous, and AA homozygous genotype.

Genomic DNA was extracted from two ml frozen EDTA-blood samples using DNA Extraction Mini Kit (YT9040, Yekta Tajhiz Azma; Iran) following the manufacturer’s instructions. rs2373929-related genotypes were typed using tetra-primer amplification refractory mutation system-polymerase chain reaction (T-ARMS PCR), while ARMS PCR was used for genotyping rs7830 polymorphism. Primers were designed using Primer 1 free online software with some modifications. The primer sequences and other important information are described in Table 1. PCR reactions were performed in the final volume of 12.5µl using a commercially PCR master mix (Yekta Tajhiz Azma, Iran) following the instruction step by step for both polymorphisms. Size differentiated amplicons were electrophoresed on 2.5% agarose gel (Figures 1 and 2).

**Table 1:** Primer sequences and their product size

**SNP**	**Primer name**	**Primer sequence (5'-3')**	**Ta (°C)**	**Amplicon size (bp)**
**rs2373929**	FO	CCCGCTGTTTCTGCTTTTC	60	FO-RO: 426 FO-RI‏ [allele T]: 307 RO-FI [allele C]: 157
	RO	TCTTTGCTCCCATTTACTGAC		
	FI	TTCCTGTGCTCTCGTGAC**C**		
	RI	GTTGCCATCTGACAGACTA**A**		
**rs7830**	FO	GACATCGCAGTCCCCTTCC	59	FO-RO: 833 Allele specific: 390
	RO	TGAGTCATCTAAGTATTCTTCAATCCAA		
	FI C	CCTTCAGGCAGTCCTTTAATC**C**		
	FI A	CTTCAGGCAGTCCTTTGGTC**A**		

SNP alleles show in bold and underline.

### Statistical analyses

Acquired data were analyzed using SPSS software v.16. Continuous values were reported as the mean ± standard deviation (SD) and compared between case and control groups by calculating independent sample *t* test. Categorical variables were presented using frequency counts and compared between groups using χ2 test. Moreover, it was used to reveal whether observed values for three different genotypes in cases and controls differ from the expected ones predicted by Hardy-Weinberg law. Logistic regression analysis was used to investigate the influence of three different genotypes of each polymorphic site on the risk of CAD by calculating the odds ratio (OR) and confidence interval (95% CI). The Dominant (comparing risk homozygous‏‏ + heterozygous vs. wild homozygous carriers) and recessive genetic model (comparing risk homozygous vs. homozygous+ wild homozygous carriers) analyses were also adopted for better interpretation of the association between the evaluated polymorphism and the CAD disease. Moreover, the influence of some probable risk factors on the susceptibility of the disease was considered by calculating OR and CI in the regression analysis. In all analysis, the p-value less than 0.05 considered significant.

## Results

### Demographic data

The demographic and clinical characteristics of the study population are summarized in Table 2. Except for hypertension, which was significantly higher in the CAD group compared to control group (*P *= 0.01), there was no significant difference in age, sex ratio (≈ 1:1), body mass index (BMI), smoking, hypercholesterolemia, diabetes mellitus, and positive family history of CAD between the patients and controls.

**Table 2:** Clinico-pathological characteristics of the study population

**Variable**	**Case (n=150)**	**Control (n=150)**	***P *** **value**	**OR (95% CI)**
Mean of age	62.09±10.31	62.18±10.8	0.71^*^	-
Mean of diagnosis age	58.98±10.63	-		-
Sex ratio ( male: female)	76:74	77:73		-
Weight	71.40±11.97	71.93±11.23	0.69	-
BMI (kg/m^2^)	26.08±4.08	26.37±3.89	0.41	-
Hypertension	104 (69.30)	83 (55.3)	**0.01** ^**^	1.83 (1.14-2.93)
Diabetes	64 (42.70)	57 (38)	0.41	1.21 (0.77-1.93)
Hypercholesterolemia	97 (64.7)	82 (54.7)	0.07	1.52 (0.95-2.41)
Smoking	37 (24.7)	23 (15.3)	**0.04**	1.81 (1.01-3.23)
Family history	16 (10.7)	25 (16.7)	0.13	0.60 (0.31-1.71)

^*^ Student t test; ^**^ Logistic regression analysis; *P* value less than 0.05 is considered significant.

### Association analyses data

#### rs2373929 polymorphism

Genotype frequencies did not deviate from Hardy–Weinberg equilibrium in the controls (χ^2^ = 2.4, *df*= 1, *P* = 0.11). Allelic and genotype distributions of the rs2373929 polymorphism are shown in Table 3. The TT homozygous genotype was more prevalent in cases compared with that in the controls (26% vs. 9.3%) and was associated with an increased risk of CAD (OR: 3.65; 95%CI: 1.77-7.53; *P* < 0.001). The allelic frequencies in the CAD group and the control group were 48% and 35% for T allele, and 52% and 65% for C allele, respectively, implying that the T-allele was associated with a greater risk of CAD (OR: 1.71; 95% CI: 1.24-2.28; *P* = 0.001). The higher risk for CAD evaluated in the recessive model for T allele (OR: 3.41; 95% CI: 1.76- 6.60; *P* < 0.001) (Table 3).

**Table 3:** Association between *ATG9B* polymorphism and risk of coronary artery disease

**SNPs**	**Variables**	**Cases**	**Controls**	***p*** ^*^	**OR (95% CI)**
	**All subjects**				
rs2373929	CC	45	59	-	1
	CT	66	77	0.65	1.12 (0.68-1.90)
	TT	39	14	**<0.001**	3.65 (1.77-7.53)
	Dominant model [TT+CT vs. CC]	105	91	0.09	1.51 (0.94-2.44)
	Recessive model [TT vs. CC+CT]	39	14	**<0.001**	3.41 (1.76-6.60)
	Allele C	156	195	-	1
	Allele T	144	105	**0.001**	1.71 (1.24-2.28)
	**Gender**				
Male	CC	21	38	**-**	1
	CT	37	34	0.06	1.97 (0.97-3.99)
	TT	18	5	**0.001**	6.51 (2.11-20.06)
	Dominant model [TT+CT vs. CC]	55	39	**0.006**	2.55 (1.30-5.00)
	Recessive model [TT vs. CC+CT]	18	5	**0.005**	4.47 (1.57-12.76)
	Allele C	79	110	**-**	1
	Allele T	73	44	**0.001**	2.31 (1.44-3.71)
Female	CC	24	21	**-**	1
	CT	29	43	0.17	0.59 (0.28-1.25)
	TT	21	9	0.15	2.04 (0.77-5.42)
	Dominant model [TT+CT vs. CC]	50	52	0.63	0.84 (0.42-1.70)
	Recessive model [TT vs. CC+CT]	21	9	**0.01**	2.82 (1.19-6.67)
	Allele C	77	85	**-**	1
	Allele T	71	61	0.29	1.29 (0.81-2.04)
	**Cholesterol level**				
Hyper	CC	32	27	-	1
	CT	37	46	0.26	0.68 (0.35-1.33)
	TT	28	9	**0.04**	2.63 (1.06-6.52)
	Dominant model [TT+CT vs. CC]	65	55	0.99	0.99 (0.53-1.86)
	Recessive model [TT vs. CC+CT]	28	9	**0.004**	3.29 (1.45-7.47)
	Allele C	101	100	-	1
	Allele T	93	64	0.09	1.44 (0.94-2.19)
Normal	CC	13	32	-	1
	CT	29	31	**0.04**	2.30 (1.01-5.23)
	TT	11	5	**0.007**	5.41 (1.57-18.68)
	Dominant model [TT+CT vs. CC]	40	36	**0.01**	2.74 (1.25-6.00)
	Recessive model [TT vs. CC+CT]	11	5	**0.03**	3.30 (1.07-10.18)
	Allele C	55	95	-	1
	Allele T	51	41	**0.005**	2.15 (1.27-3.65)
	**Genotypes**				
rs7830	CC	55 (36.7)	57 (38)	-	Reference
	AC	74 (49.3)	74 (49.3)	0.88	1.03 (0.63-1.69)
	AA	21 (14)	19 (12.7)	0.71	1.14 (0.55-2.36)
	**Alleles**				
	C	183 (0.61)	188 (0.63)	-	reference
	A	116 (0.39)	112 (0.37)	0.74	1.06 (0.77-1.47)

Logistic regression analysis; *P* value less than 0.05 is considered significant.

In the next step, the data were stratified according to gender and cholesterol level. A significantly increased risk of CAD was shown in all genetic models in male (*P* < 0.05), while the similar result acquired only in a recessive model in female (OR: 2.82; 95% CI: 1.19-6.67; *P* = 0.01) (Table 3).

The frequency of TT genotype was significantly higher in cases compared to controls in hypercholesterolemia subgroup implying the greater risk of CAD (OR: 2.63; 95%CI: 1.06-6.52; *P* = 0.04). The increased risk of CAD was seen in the recessive model for T allele (OR: 3.29; 95%CI: 1.45-7.47; *P* = 0.004). In a normal-cholesterol subgroup, an increased risk of disease was observed in the co-dominant model as well as the recessive and dominant model for T allele (Table 3). Higher frequency of T allele was evaluated in cases of normal-cholesterol subgroup (OR: 2.15; 95% CI: 1.27- 3.65; *P*= 0.005).

Further analysis using a logistic regression model, adjusting the data for known risk factors for CAD including age, sex, BMI, hypertension, hypercholesterolemia, diabetes, family history, and smoking, the TT genotype showed strong association with increased risk of CAD in co-dominant (OR: 3.70; 95% CI: 1.33-7.91; *P* = 0.001) and recessive (OR: 3.42; 95% CI: 1.72-6.83; *P* < 0.001) genetic mode of inheritance. Moreover, hypertension and smoking seem to be independent risk factors for disease susceptibility, while hypercholesterolemia increased the risk of CAD only in dominant (OR: 1.68; 95% CI: 1.02-2.76; *P* = 0.04) genetic model (Table 4).

**Table 4:** Influence of rs2373929 variant on the risk of CAD after adjustment for potential variables

**Risk factors**	**Co-dominant model**		**Dominant model**		**Recessive model**	
	***P***	**OR (95% CI)**	***P*** ^*^	**OR (95%CI)**	***P***	**OR (95%CI)**
Sex	0.66	1.13 (0.66-1.94)	0.53	1.18 (0.70-2.00)	0.64	1.14 (0.66-1.94)
Age (yr)	0.55	0.99 (0.97-1.02)	0.67	0.99 (0.97-1.19)	0.52	0.99 (0.97-1.02)
BMI (kg/m^2^)	0.63	0.96 (0.93-1.05)	0.53	0.98 (0.92-1.04)	0.66	0.99 (0.93-1.05)
Hypertension	**0.002**	2.36 (1.37-4.08)	**0.002**	2.34 (1.37-3.98)	**0.002**	2.39 (1.40-4.11)
Diabetes	0.09	1.55 (0.93-2.58)	0.12	1.49 (0.89-2.46)	0.09	1.54 (0.93-2.60)
Hypercholesterolemia	0.07	1.59 (0.96-2.65)	**0.04**	1.68 (1.02-2.76)	0.07	1.60 (0.97-2.66)
Smoking	**0.01**	2.51 (1.25-5.05)	**0.009**	2.50 (1.26-4.98)	**0.01**	2.49 (1.24-5.03)
Family history	0.09	0.54 (0.27-1.11)	0.08	0.54 (0.26-1.09)	0.10	0.55 (0.27-1.12)
rs2373929	**0.001**	3.70 (1.33-7.91)	0.10	1.53 (0.92-2.54)	**<0.001**	3.42 (1.72-6.83)

^*^Multiple logistic regression model; Co- dominant model: TT CC; Dominant model: TT+CT vs. CC; Recessive model: TT vs. CC+CT; BMI: Body mass index

#### rs7830 polymorphism

The genotype distributions in control population was in accordance with expected frequencies in Hardy–Weinberg equilibrium (χ^2^ = 0.24, *df*= 1, *P* = 0.06). The data related to the association analysis of this polymorphism with CAD are presented in Table 3. The genotype frequencies were the same in cases and controls, therefore, no association was found between any genotypes of this polymorphism and CAD. The frequency of the A-allele was 37% and 39% in control and cases, respectively. The difference in the allelic frequencies was not statistically significant.

## Discussion

LncRNAs are the newly described transcripts, however, their simultaneous emergence with the strong demand for finding out diagnostic molecular markers in medicine attracted lots of attention to these novel long transcripts. Using association studies, the relationship between several SNPs in different types of lncRNAs was investigated. In this regard, one of the first studies was by Ishii et al which identified and described the functional long non-coding RNA, called myocardial infarction antisense transcript (*MIAT*). They believed that the differential expression of MIAT in the presence of different alleles of rs2331291 polymorphism plays a role in MI pathogenesis.^19^ In further analysis, an increased risk of MI was seen in the presence of a susceptible allele of the rs1333049 polymorphism in ANRIL lncRNA gene.^20^ Gao et al demonstrated the relationship between H19 lncRNA and the susceptibility to CAD in a Chinese population.^21^ In a year after, Huang et al examined the association between the common variant in CDKN2BAS lncRNA and coronary heart disease. Their findings supported the relationship between this lncRNA and coronary heart disease, especially in patients younger than 65 years. They validated this result by performing a meta-analysis.^22^ Recently, Tang et alhave shown decreased risk of CAD in the presence of G-A-A-G haplotype of the risk alleles of *lincRNA-p21* gene rs9380586, rs4713998, rs6930083, and rs6931097 polymorphisms in Chinese population.^23^

We were interested to consider the *ATG9B* gene for further investigation because of the valuable achievements regarding the biological role of ATG9B in post-transcriptional regulation of eNOS gene expression.^12^ eNOS is responsible for the physiological availability of NO and is the second most considered gene in the pathogenesis of CAD.^24^ The sense/antisense interaction of *ATG9B*/eNOS is functionally important and has been shown to participate in vascular disease pathogenesis.^12^ Since variations in *ATG9B* gene may interfere with ATG9B-eNOS interaction and alter the gene expression or splicing pattern, in the present study, we investigated the possible association of *ATG9B* rs2373929 and rs7830 gene polymorphisms with the risk of CAD in a population from southwest Iran. Our statistical analyses provided information in favor of an increase in the risk of CAD in subjects with TT genotype for rs2373929 polymorphism. In the pooled-based genome-wide association analysis, Sebastiani et al examined the functional significance of thousands of SNPs as well as rs2373929; they found this variant as a potential marker for longevity.^25^ Carreras-Torres et al had shown the association between the T allele of rs2373929 polymorphic site and risk of MI.^26^ rs2372929 is the intronic variant that appears to alter the proper splicing and subsequently reduce the stability of the lncRNA. The NCBI database introduces the benign clinical significance for rs7830 polymorphism. As far as we know, this is the first study investigating the effect of this variant on the predisposition to CAD. As our results showed, genotypes had the same frequencies in cases and controls. Our results are in contradiction with the finding of Li et al. They reported an increased risk of hypertension among the rs7830T allele carriers.^27^ In another research, Jimenez-Sousa and colleagues found rs7830T allele a significant risk factor for end-stage renal disease.^28^

In the present study, the sample size was quite small and further investigations enrolling a large number of subjects suggested to validate the results. One of the other limitations of our work was that we did not investigate the association of the *ATG9B* gene polymorphisms and severity of the disease regarding the number of diseased vessels. Moreover, we did not perform any clinical evaluation for recruiting control group subjects then it leads to uncertainty about the healthiness of the control group and subsequently the results may be biased. The level of triglyceride, total cholesterol, high-density cholesterol, and low-density cholesterol should be accounted and the association of the *ATG9B* polymorphisms with these factors should be researched.

## Conclusion

In conclusion, the *ATG9B* rs2373929 polymorphism might be considered as a molecular marker in screening high-risk individuals for CAD.

## Ethical Approval

The study was approved by the local ethics committee in our department.

## Competing interests

The authors report no conflicts of interest.

## Acknowledgments

The authors give their special thanks to the staff of the Al-Zahra heart hospital angiography ward for their collaboration in blood sampling. The study was partially funded by Islamic Azad University, Arsanjan Branch.
